# Comparative analysis of jejunal morphology, antioxidant capacity and barrier function between Dahe pigs and Dahe black pigs

**DOI:** 10.3389/fvets.2026.1772556

**Published:** 2026-05-13

**Authors:** Huijin Jia, Wenzhe Shi, Shiqi Xue, Wanghong Zhang, Guangyao Song, Lanlan Yi, Junhong Zhu, Sumei Zhao

**Affiliations:** Faculty of Animal Science and Technology, Yunnan Agricultural University, Kunming, China

**Keywords:** antioxidant capacity, barrier function, gene expression, jejunal morphology, pigs

## Abstract

This research systematically compared the jejunum morphology, antioxidant capacity, barrier-related gene expression, and mucosal immune status between two Yunnan local pig breeds, namely Dahe pigs (DH) and Dahe black pigs (DHB), using histopathological staining, antioxidant assays, quantitative polymerase chain reaction (qPCR), immunofluorescence, and enzyme—linked immunosorbent assay (ELISA). The outcomes indicated that DHB presented a shallower crypt depth and a higher ratio of villus height to crypt depth, suggesting an enhanced capacity for nutrient absorption. In their jejunum, the activities of glutathione (GSH), superoxide dismutase (SOD), and catalase (CAT) were significantly elevated, whereas the levels of malondialdehyde (MDA) were notably reduced. Conversely, DH displayed superior barrier function, with significantly higher expression levels of tight-junction proteins (Occludin, *OCLN;* Claudin-1, *CLDN1* and Claudin-2, *CLDN2)*, mucin genes (*MUC1*), defense-related proteins (*DEFB1, DEFB2*), and immune-related genes (Interleukin-10, *IL-10;* Toll-like receptor 4, *TLR4;* Myeloid differentiation factor 88, *MyD88;* Immunoglobulin heavy chain A, *IGHA;* Joining chain, *JCHAIN* and Polymeric immunoglobulin receptor, *PIGR*), along with a significant increase in IgG levels (*p* < 0.05). These findings uncover the distinct physiological and immunological characteristics between the two breeds, offering theoretical underpinnings for precision nutrition and healthy breeding strategies in local pig populations.

## 1 Introduction

The jejunum, as a key segment of the small intestine, plays a critical role in nutrient absorption and intestinal homeostasis, with its structural integrity, antioxidant capacity, and barrier function directly affecting the health and production performance of pigs (1, 2). Oxidative stress and intestinal barrier dysfunction can disrupt nutrient utilization and induce inflammatory responses, while the intestinal mucosal immune system further safeguards the host against pathogenic invasion (3, 4).

Dahe pigs (DH) and Dahe black pigs (DHB) are valuable local pig breeds in Yunnan Province, China. DH is renowned for its excellent maternal traits, strong stress resistance, and high intramuscular fat content, while DHB, a lean-type breed developed by introducing Duroc half-blood into DH, retains advantages such as rapid growth, good meat quality, and tolerance to coarse feed (5, 6). Currently, research on these two breeds has predominantly focused on muscle growth, development, and fat deposition (7–12), with relatively scarce investigations into intestinal health-related traits, including jejunal morphology, antioxidant capacity (based on total antioxidant capacity, T-AOC; glutathione, GSH; superoxide dismutase, SOD; catalase, CAT and malondialdehyde, MDA), and barrier function (encompassing physical, chemical, and immune barriers). Given the crucial role of intestinal function in nutrient absorption and disease resistance, exploring the differences in jejunal physiological and immunological characteristics between DH and DHB is essential for optimizing their breeding strategies and nutritional management.

To address this research gap, the present study employed histopathological staining, antioxidant capacity assays, quantitative polymerase chain reaction (qPCR), immunofluorescence, and enzyme-linked immunosorbent assay (ELISA) techniques to systematically compare jejunal morphology (villus height, crypt depth, goblet cell number), antioxidant indexes (T-AOC, GSH, SOD, CAT, MDA), barrier-related gene expression (tight junction proteins, mucins, defensins), and mucosal immune status (immunoglobulins) between DH and DHB. The aim was to uncover the distinct intestinal functional characteristics of the two breeds, thereby providing theoretical support for precision nutrition and healthy breeding of local pig populations.

## 2 Materials and methods

### 2.1 Experimental animals and sample collection

In this study, both DH and DHB were uniformly raised and managed by the DH Breeding Farm (core population farm) in Fuyuan County, Yunnan Province. A total of 180 pigs, consisting of 90 DH and 90 DHB, with a body weight of 30.00 ± 2.54 kg, were selected. The pigs of each breed were in good condition and were evenly divided into males and females. Each pig was housed in a separate stall and was fed the same basic diet, with free access to feed and water in identical environments. The pig house was maintained with scientifically optimized environmental parameters, including a temperature controlled within 18–26 °C, humidity regulated between 60%−80%, and a stocking density complying with industry standards of 1.1–1.3 m2/head for finishing pigs. Through comprehensive ventilation systems and spatial layout design, all pens was achieved uniform temperature–humidity distribution with variations maintained within ± 2 °C and ± 5% RH, respectively. This controlled environment was ensured thermal comfort and air quality consistency (NH_3_ < 20 mg/m^3^, H_2_S < 10 mg/m^3^) across all animal zones. Upon reaching a weight of 120 ± 6.83 kg, 6 pigs (half male and half female) were randomly selected for slaughter in two groups. Jejunal tissues and contents were collected and placed into 1.5 ml frozen storage tubes then stored in liquid nitrogen for subsequent analysis. The research proposal and related experimental procedures had been approved by the Animal Care and Use Committee of Yunnan Agricultural University (Case Number: 20210915). Written informed consent was obtained from the owners for the participation of their animals in this study.

### 2.2 Test diet

The dietary formula for this experiment was designed in accordance with the nutritional requirements for local meat-fat dual-purpose pig breeds, as specified in the pigs feeding standard using http://down.foodmate.net/standard/sort/3/91362.html (accessed on 10 May 2024). Diets for different weight stages (30–60 kg and 60–120 kg) were prepared. The dietary composition and nutritional levels were detailed in Table 1.

**Table 1:** Composition and nutrient levels of basal diets (air-dry basis).

Item	BW (kg)	
	30~60	60~120
Raw material (%)		
Corn	64.21	61.50
Soybean meal	16.11	15.00
Rice bran	10.00	10.00
Wheat bran	7.10	10.00
Stone powder	1.08	2.00
Premix	1.00	1.00
Calcium hydrogen phosphate	0.32	0.00
Salt	0.18	0.50
Total	100	100
Nutrient level		
pigs digestive energy (MJ/kg)	13.81	13.51
Crude protein^*^	15.17	14.85
Lysine^*^	0.63	0.55
Calcium^*^	0.55	0.81
Total phosphorus^*^	0.53	0.49

^*^Measured value.

(1) Meat fat type growth and finishing pigs (30~60kg) premix provides: Vitamin A 5,000 IU, Vitamin D_3_ 180 IU, Vitamin E 15 mg, Vitamin K_3_ 0.50 mg, Thiamine Vitamin B_1_ 1.50 mg, Riboflavin Vitamin B_2_ 2.50 mg, Niacin VPP 12.00 mg, Pantothenic acid B_5_ 9.00 mg, Pyridoxine B_6_ 1.00 mg, biotin VH 0.08 mg, folic acid 0.30 mg, vitamin B_12_ 10.00 mg, choline 350 mg, iron 148.34 mg, copper 199.50 mg, manganese 113.50 mg, zinc 65.00 mg, iodine 0.15 mg, selenium 0.25 mg. Meat fat type growth and finishing pigs (60~100 kg) provides: Vitamin A 5,000 IU, Vitamin D_3_ 160 IU, Vitamin E 15 mg, Vitamin K_3_ 0.50 mg, Thiamin B_1_ 1.50 mg, Riboflavin B_2_ 2.50 mg, Niacin Vpp 9.00 mg, Pantothenic acid B_5_ 8.00 mg, Pyridoxine B_6_ 1 mg, Biotin VH 0.08 mg per kg of diet. Folic acid 0.30 mg, vitamin B_12_ 6.00 mg, choline 0.40 g; iron 258.56 mg, copper 199.50 mg, manganese 113.50 mg, zinc 55.00 mg, iodine 0.15 mg, selenium 0.25 mg. (2) ^*^ is the measured value.

### 2.3 Paraffin sectioning and staining methods

Jejunal paraffin sections were prepared by trimming the fixed jejunal tissues and placing them into paraffin embedding cassettes for dehydration, clearing, wax infiltration and embedding. After embedding, the paraffin blocks were transferred to a 4 °C refrigerator for accelerated solidification and subsequent storage. The embedded tissues were sectioned with a microtome to obtain intact tissue sections. Following sectioning, the tissue sections were subjected to slide mounting and air-drying, and then processed sequentially with the following procedures: dewaxing, hematoxylin staining, eosin staining, dehydration, and clearing. Finally, the stained sections were mounted with neutral mounting medium.

### 2.4 Determination of intestinal antioxidant index

0.1 g of thawed jejunal tissue was weighed, 0.9 ml of pre-chilled homogenization buffer was added, and homogenization was performed under ice bath conditions. The mixture was centrifuged at 7,500 rpm and 4 °C for 10 min for the collection of the supernatant. Jejunal total antioxidant capacity (T-AOC), glutathione (GSH) content, superoxide dismutase (SOD) activity, catalase (CAT) activity and malondialdehyde (MDA) content in DH and DHB were determined using commercial assay kits provided by the Nanjing Institute of Bioengineering and Biotechnology. All assay procedures were performed in accordance with the manufacturers' kit instructions: the optical density (OD) values of T-AOC, GSH and SOD were detected via enzyme immunoassay (EIA), while the absorbance values of CAT and MDA were determined by spectrophotometry. The total protein concentration in homogenate supernatants was quantified using the Bradford brilliant blue method, and all experimental measurements were calculated in accordance with the corresponding standard formulas.

### 2.5 Determination of intestinal barrier related gene expression

Total RNA was extracted from porcine jejunal tissue in accordance with the manufacturer's protocol for TRI Reagent (a total RNA extraction reagent, Biosharp, China). The qualified total RNA extracted from jejunal tissue was reverse-transcribed into complementary DNA (cDNA) using the PrimeScript™ RT Reagent Kit with gDNA Eraser (TaKaRa, China). The mRNA sequences of the target genes used in this experiment were obtained from the National Center for Biotechnology Information (NCBI), and the GAPDH gene was used as the reference gene for data normalization. Gene-specific primers required for the experiment were designed using Primer-BLAST and subsequently synthesized by Tsingke Biotech (China) (Table 2). Quantitative real-time polymerase chain reaction (qPCR) was performed using the Tli RNaseH Plus Fluorescent Quantitative PCR Kit (TransGen, China, RR820A), with a total reaction volume of 20 μL. The reaction system consisted of 0.4 μL of each forward and reverse PCR primer, 10 μL of 2 × PerfectStart Green qPCR SuperMix, 7.7 μL of nuclease-free water, and 1.5 μL of cDNA template. The qPCR reaction was carried out on a real-time PCR instrument (Bio-Rad, USA) under the following cycling conditions: an initial denaturation at 94 °C for 3 min, followed by 44 cycles of denaturation at 94 °C for 5 s, annealing at 52.8 °C for 15 s, and extension at 72 °C for 10 s. The relative expression levels of the target genes were calculated using the 2^∧−ΔΔ*Ct*^ method, with *GAPDH* as the reference gene for data normalization. Each sample was analyzed in triplicate to ensure experimental repeatability, and the results were presented as the mean ± standard deviation (SD).

**Table 2:** The primer sequence, annealing temperature of target gene and internal reference gene.

Genes	Primer sequences (5^′^-3^′^)	Annealing temperature
*GAPDH*	F: CCCACGGAATCGAGAAAGAG	56.5 °C
	R: TGGTGCCCTTCCGTCAA	
*DEFB1*	F: TGCCACAGGTGCCGATCT	58.5 °C
	R: CTGTTAGCTGCTTAAGGAATAAAGGC	
*DEFB2*	F: CCAGAGGTCCGACCACTACA	60 °C
	R: GGTCCCTTCAATCCTGTTGAA	
*MUC1*	F: GTGCCGCTGCCCACAACCTG	58.5 °C
	R: AGCCGGGTACCCCAGACCCA	
*MUC2*	F: GGTCATGCTGGAGCTGGACAGT	61 °C
	R: TGCCTCCTCGGGGTCGTCAC	
Tight junction protein 1*, TJP-1*	F: CAGAGACCAAGAGCCGTCC	60 °C
	R: TGCTTCAAGACATGGTTGGC	
Tight junction protein 2*, TJP-2*	F: CAGCCGTGAGCACCTCC	60 °C
	R: GCCGGAGACCATACTCTTCA	
*OCLN*	F: TCAGGTGCACCCTCCAGATT	61 °C
	R: AGGAGGTGGACTTTCAAGAGG	
*CLDN1*	F: ATTTCAGGTCTGGCTATCTTAGTTGC	61 °C
	R: AGGGCCTTGGTGTTGGGTAA	
*CLDN2*	F: TGGTGCCTGACAGCATGAAA	61 °C
	R: GGGCCTGATAGGCATCGTAG	
*IGHA*	F: CTGGTGACACTGACATGCCT	60 °C
	R: TACTTGTCACGGGGCAGTTC	
*JCHAIN*	F: CTGTTTTTGTGACAGCCCAAG	59.5 °C
	R: GGAAGTAATCCGGGCACACT	
*PIGR*	F: AGCCAACCTCACCAACTTCC	60 °C
	R: CTGCTAATGCCCAGACCAC	
Cluster of differentiation 14, *CD14*	F: CGTTTGTGGAGCCTGGAAG	60 °C
	R: TGCGGATGCGTGAAGTTG	
Cluster of differentiation 40, *CD40*	F: ATCCTGTTTGCCGTCCTGTT	60 °C
	R: CTTAGGGTGCAGGGCCTTAG	
*IL-10*	F: GCTGAAGACCCTCAGGCTGA	59 °C
	R: TTGCTCTTGCTTCTCCACT	
*TLR4*	F: ACCAGACTTTCTTGCAGTGGGTCA	51.5 °C
	R: AATGACGGCCTCGCTTATCTGACA	
*MyD88*	F:TCATGTTCTCCATACCCTTGGT	60 °C
	R:AAACTGCGAGTGGGGTCAG	

### 2.6 Immunofluorescence

The samples were soaked in xylene I for 5 min, xylene II for 3 min, 100% alcohol for 1 min, 95% alcohol for 2 min, 85% alcohol for 1 min, and finally in distilled water for 1 min. The slides were immersed in 0.01 M Tris-EDTA antigen retrieval solution (pH 9.0) (G1218, Servicebio, Wuhan, China), and were subjected to microwave-assisted antigen retrieval after the slanted lid was placed on the container (high power for 3 min followed by static incubation for 5 min, repeated once; medium-low power for 1 min followed by static incubation for 5 min). After cooling to room temperature, 5% normal goat serum (GC305010, Servicebio, Wuhan, China) was applied to the samples and was incubated at 37 °C for 30 min in a humidified chamber. Primary antibody incubation: The primary antibody working solution (GB21303, Servicebio, Wuhan, China) was prepared by the dropwise addition of TBS buffer (G4202, Servicebio, Wuhan, China), and was added dropwise to the samples and incubated overnight at 4 °C in a humidified chamber. Re-warming: The slides were allowed to rewarm at room temperature for 15 min; subsequently, the primary antibody solution was discarded, and the slides were washed once with Tris-buffered saline with Tween-20 (TBST), followed by three washes with TBST (5 min each). Secondary antibody incubation: The corresponding fluorescent secondary antibody working solution (GC305010, Servicebio, Wuhan, China) was added dropwise, and was incubated at 37 °C in the dark for 1 h. After discarding the secondary antibody solution, the slides were washed once with TBST, followed by three washes with TBST (5 min each) 0.4′,6-Diamidino-2-phenylindole (DAPI) working solution (G1012, Servicebio, Wuhan, China) was added dropwise, was incubated at room temperature in the dark for 10 min; then the DAPI solution was discarded, and the slides were washed once with TBST, followed by three washes with TBST (5 min each). Finally, anti-fade mounting medium (G1401, Servicebio, Wuhan, China) was added dropwise to the slides, and the slides were sealed with coverslips for observation and imaging under a fluorescence microscope. Standardized image processing was performed using ImageJ software (v1.54f): background subtraction was applied with a rolling ball radius of 80 pixels, followed by brightness/contrast calibration using identical minimum and maximum grayscale values for all images. No group-specific adjustments were made to preserve the relative differences in signal intensity between DH and DHB groups.

### 2.7 ELISA assay

Commercial ELISA kits (Enzyme-linked Bio, Shanghai, China) were used to determine the concentrations of IgA, SIgA, IgM, and IgG in the jejunal tissues of DH and DHB. The concentrations of the target proteins were quantified against a standard curve.

### 2.8 Data processing

The experimental data were collated using Microsoft Excel 2016. Unpaired Student's *t-*tests were conducted to analyze the results of jejunal HE staining, PAS staining, antioxidant index assays, ELISA, immunofluorescence staining, and qPCR assays for DH and DHB, using IBM SPSS Statistics 23.0 software. The significance level was set at *p* < 0.05. The normality of the data was verified by the Shapiro–Wilk test (α = 0.05), and the homogeneity of variance was tested by Levene's test. All datasets were tested for normality and homogeneity of variance, and all tests yielded *p*-values > 0.05, indicating that the assumptions for parametric testing were met. Since all data satisfied the assumptions of parametric tests, non-parametric statistical methods were not employed. Intergroup comparisons of data that met the parametric test assumptions were performed using unpaired Student's *t*-tests. All experimental data were expressed as the mean ± standard error of the mean (SEM), where *p* < 0.05 was considered to indicate a significant difference and *p* < 0.01 was considered to indicate an extremely significant difference. Each gene expression assay was performed in triplicate, and histograms of the gene expression results were plotted using GraphPad Prism 9.0 (GraphPad Software, USA).

## 3 Results

### 3.1 Analysis of intestinal morphology and structure

**Figure 1:**
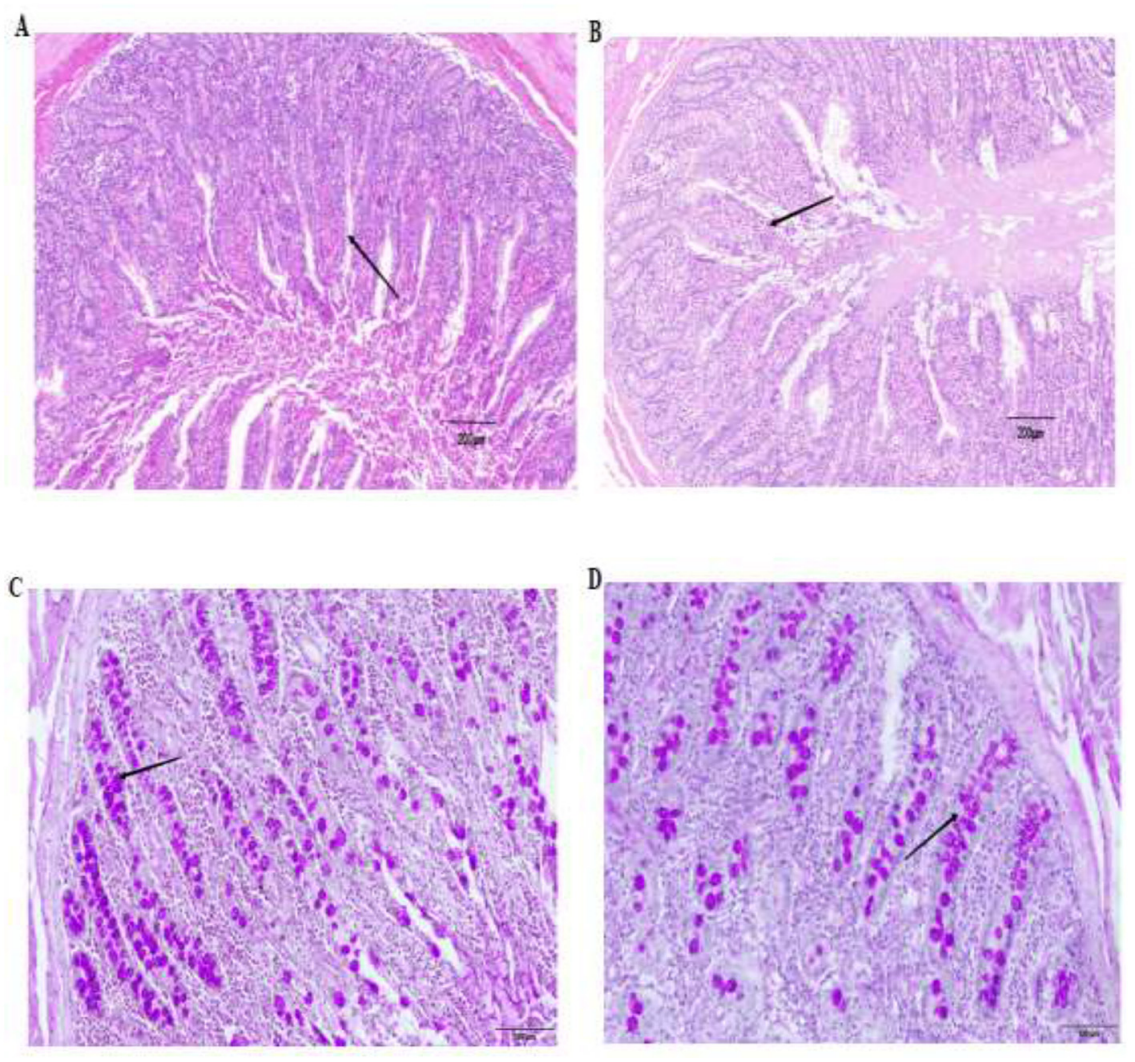
Jejunum villus morphology and goblet cell morphologyof DH and DHB (×50). The arrow points to intestinal villi and goblet cells of the jejunum. **(A)** Jejunum villus morphology of DH; **(B)** Jejunum villus morphology of DHB; **(C)** Goblet cell morphology of jejunum of DH; **(D)** Goblet cell morphology of jejunum of DHB.

Morphological analysis of the jejunum indicated that the epithelial cells in the DH group were densely packed, with deep and narrow crypt spaces. In contrast, the epithelial cells in the DHB group were arranged relatively loosely, featuring shallow and wide crypt spaces (Figures 1A, B). The crypt depth in the DHB group (143.79 ± 31.48 μm) was significantly lower compared to that in the DH group (184.61 ± 25.29 μm), and the V/C ratio (3.66 ± 0.43) was significantly higher than that in the DH group (2.39 ± 0.52) (*p* < 0.05) (Table 3). Statistical analysis of the jejunal goblet cells demonstrated that the quantity of goblet cells in the DHB group was significantly less than that in the DH group (*p* < 0.05) (Figures 1C, D; Table 3).

**Table 3:** Intestinal morphological structure and goblet cell number in jejunum of DH and DHB.

Site	DH groups	DHB group
Villus height/μm	432.38 ± 68.69	496.55 ± 60.73
Crypt deepth/μm	184.61 ± 25.29^a^	143.79 ± 31.48^b^
Villus height/crypt depth	2.39 ± 0.52^a^	3.66 ± 0.43^b^
Goblet cell/number	35.32 ± 3.24^a^	26.61 ± 1.99^b^

Different lowercase letters represent a significant difference (*p* < 0.05).

### 3.2 Antioxidant index of jejunum tissue

Compared with DH group, GSH activity, SOD activity and CAT activity of jejunal tissue in DHB group were significantly higher than DH group (*p* < 0.05), and MDA content was significantly lower than DH group (*p* < 0.05), but T-AOC between the two groups showed no significant difference (*p* > 0.05), as shown in Table 4.

**Table 4:** Antioxidant capacity of jejunum tissues of DH and DHB.

Item	DH groups	DHB group
T-AOC (mM)	1.02 ± 0.22	0.98 ± 0.16
GSH (nmol/mgprot)	37.86 ± 4.50^a^	62.74 ± 3.94^b^
SOD (U/mgprot)	5.05 ± 0.69^a^	11.57 ± 1.42^b^
CAT (U/mgprot)	2.73 ± 0.45^a^	6.21 ± 1.10^b^
MDA (nmol/mgprot)	9.64 ± 1.30^a^	3.12 ± 0.64^b^

Different lowercase letters represent a significant difference (*p* < 0.05).

### 3.3 ELISA assay

Compared with DH group, the content of IgG in jejunal tissue of DHB group was significantly lower than that of DH group (*p* < 0.05), and there was no significant difference in Secretory immunoglobulin A (SIgA), Immunoglobulin M (IgM) and Immunoglobulin A (IgA) content between the two groups (*p* > 0.05), as shown in Table 5.

**Table 5:** Test results of jejunum tissue immune indexes of DH and DHB.

Item	DH groups	DHB group
IgA (μg/ml)	16.54 ± 2.10	15.30 ± 1.83
SIgA (μg/ml)	0.83 ± 0.06	0.79 ± 0.02
IgM (mg/ml)	0.47 ± 0.07	0.60 ± 0.09
IgG (mg/ml)	0.69 ± 0.18^a^	0.39 ± 0.10^b^

Different lowercase letters represent a significant difference (*p* < 0.05).

### 3.4 mRNA expression levels of genes involved in intestinal tissue barrier function

**Figure 2:**
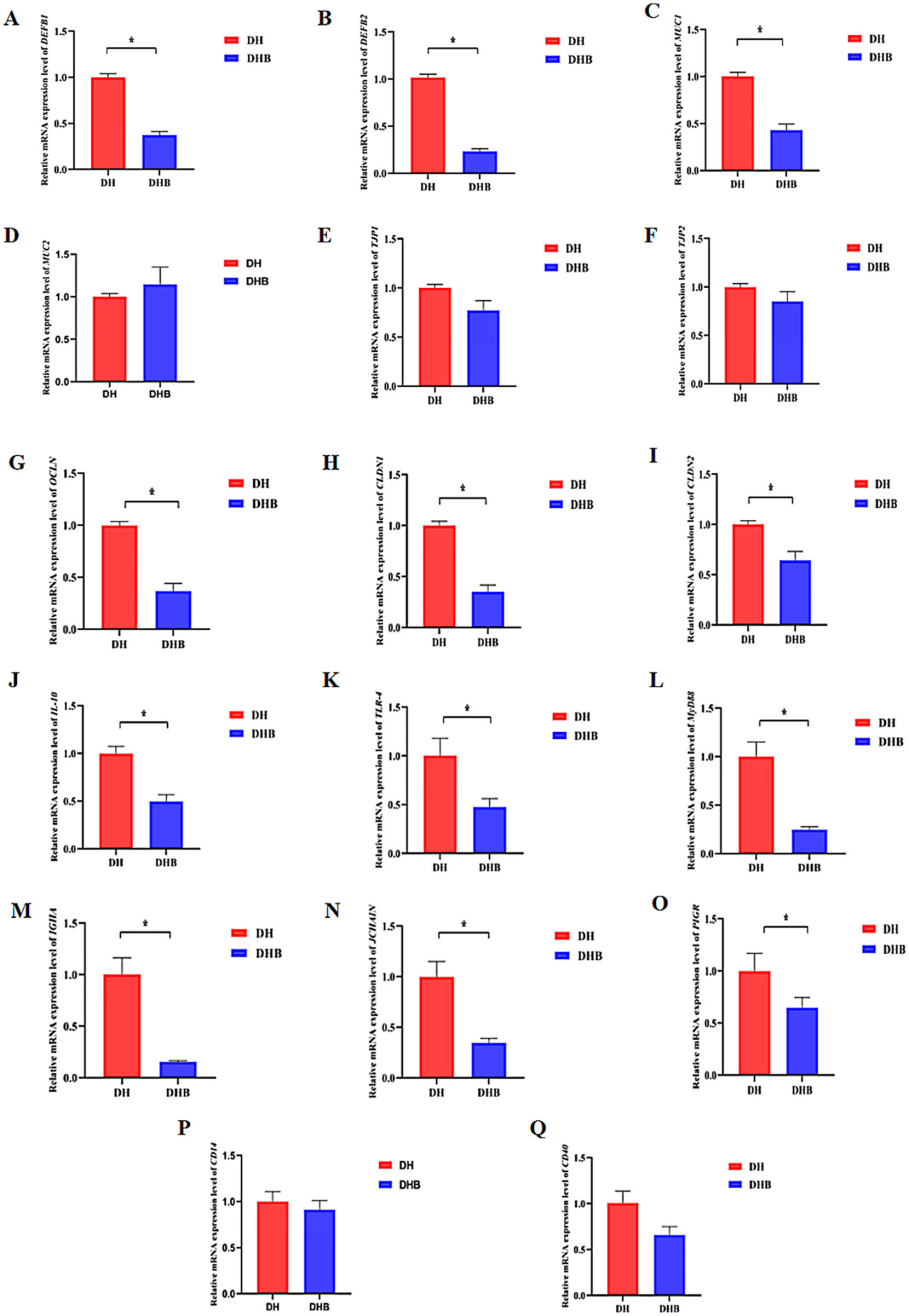
Relative expression of mRNA of genes related to intestinal barrier function in DH and DHB. ^*^indicates a significant difference (*p* < 0.05). **(A)** *DEFB1* gene; **(B)** *DEFB2* gene; **(C)** *MUC1* gene; **(D)** *MUC2* gene; **(E)** *TJP1* gene; **(F)** *TJP2* gene; **(G)** *OCLN* gene; **(H)** *CLDN1* gene; **(I)** *CLDN2* gene; **(J)** *IL-10* gene; **(K)** *TLR4* gene; **(L)** *MyD88* gene; **(M)** *IGHA* gene; **(N)** *JCHAIN* gene; **(O)** *PIGR* gene; **(P)** *CD14* gene; **(Q)** *CD40* gene.

The mRNA expression of *DEFB1, DEFB2, MUC1, OCLN, CLDN1, CLDN2, IL-10, TLR4, MyD88, IGHA, JCHAIN*, and *PIGR* genes in DHB group was significantly lower than that in DH group (*p* < 0.05), while the mRNA expression of *MUC2, TJP1, TJP2, CD14*, and *CD40* genes showed no significant difference between the two groups (*p* > 0.05). The results are shown in Figure 2.

### 3.5 Analysis of jejunal immunofluorescence results

**Figure 3:**
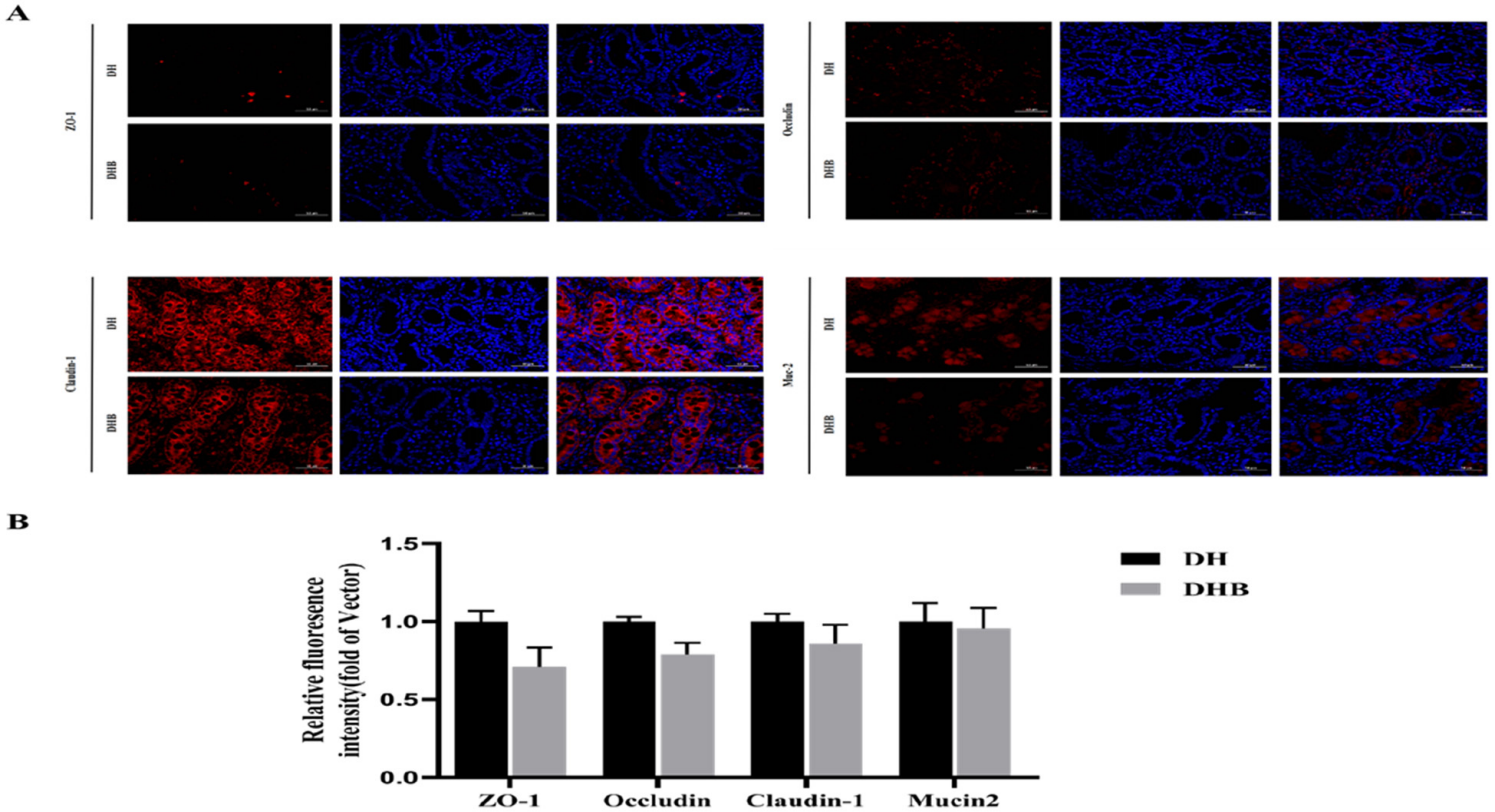
Immunofluorescence staining of jejunal tissues in DH and DHB (×50). **(A)** Immunofluorescence images of ZO-1, Occludin, Claudin-1 and Mucin2 (red: target protein; blue: DAPI-stained nucleus; scale bar = 200 μm); **(B)** Semi-quantitative analysis of positive signal area ratio and mean optical density of target proteins. ns, no significant difference (*p* > 0.05).

The red area showed positive results for ZO-1, Occludin, Claudin-1 and Mucin2 (Figure 3). Compared with the DH group, the DHB group showed relatively lower expression levels of ZO-1, Occludin, Claudin-1, and Mucin2, but the difference was not significant (*p* > 0.05).

## 4 Discussion

Food traverses from the oral cavity through the stomach to the small intestine, where it is subjected to precise regulation and digested into smaller molecules such as amino acids, glucose, and fatty acids. These nutrients are absorbed via the mucosa and promptly enter the circulatory system through microvessels and chyle. The primary physiological function of the jejunum is to digest and absorb nutrients, thereby providing essential nourishment for the host (2). The complete digestion and absorption of nutrients rely on the structural integrity of the jejunum, the intestinal epithelium of which is composed of numerous self - renewing crypt—villus axis units (13). Volumetric villus length (V), crypt depth (C), and the villus—to—crypt ratio (V/C) act as indicators of intestinal epithelial cell proliferation, which impacts nutrient absorption and transport in the jejunum (14–16). The jejunum harbors countless villi, each possessing numerous microvilli (17). Intestinal crypts are invaginations in the intestinal wall. An increase in crypt depth diminishes nutrient absorption capacity, and the V/C ratios are positively correlated with animal growth rates (18). Longer villi and shallower crypts signify stronger digestive and absorptive capabilities in the jejunum (19, 20). In this study, DHB exhibited significantly shallower crypt depth and higher V/C ratio compared to DH, coupled with significantly elevated activities of antioxidant enzymes (GSH, SOD, CAT) and reduced MDA content. This dual advantage of “superior nutrient absorption + enhanced antioxidant capacity” in DHB is not a random combination but a genetically driven functional specialization shaped by breeding selection. As a lean-type breed derived from DH crossed with Duroc half-blood (5, 6), DHB inherited the rapid growth trait of Duroc pigs (21). Rapid muscle growth and lean meat deposition require efficient nutrient uptake and robust redox homeostasis to mitigate oxidative stress induced by high metabolic rates (9). The upregulated antioxidant enzymes in DHB's jejunum likely form a protective network: SOD catalyzes the dismutation of superoxide anions into hydrogen peroxide, CAT further decomposes hydrogen peroxide into water and oxygen, and GSH scavenges free radicals and maintains intracellular redox balance (22). This synergistic antioxidant system prevents lipid peroxidation (evidenced by lower MDA) and protects intestinal epithelial cell membranes from oxidative damage, thereby preserving the structural integrity of the villus-crypt axis and sustaining efficient nutrient absorption. Consistent with this, previous studies on Duroc-derived hybrids have shown that enhanced growth performance is often accompanied by improved intestinal antioxidant capacity (23), confirming that the functional coupling of absorption and antioxidation in DHB is a adaptive trait associated with its lean-type genetic background.

In contrast, DH, as a native local breed, displayed superior intestinal barrier and immune function, characterized by significantly higher expression levels of tight junction proteins genes (*OCLN, CLDN1, CLDN2*), mucin (*MUC1*), defensins (*DEFB1, DEFB2*), and immune-related genes (*IL-10, TLR4, MyD88, IGHA, JCHAIN, PIGR*), as well as elevated IgG content. This comprehensive barrier advantage reflects the evolutionary adaptation of local breeds to complex environmental conditions. Native pig breeds typically evolve robust intestinal defense systems to resist pathogenic microorganisms and environmental stressors in their indigenous habitats (24). The tight junction proteins genes (*OCLN, CLDN1, CLDN2*) form a physical barrier that reduces intestinal permeability and prevents the translocation of luminal antigens and pathogens (25, 26); *MUC1* and defensins (*DEFB1, DEFB2*) constitute a chemical barrier by enhancing mucus layer stability and exerting antimicrobial effects (27, 28); while *IL-10, TLR4*/*MyD88* signaling pathway, and immunoglobulins (IgG, IgA) regulate mucosal immune responses—*IL-10* inhibits excessive inflammation and maintains epithelial homeostasis (29, 30), TLR4/MyD88 mediates pathogen recognition and immune activation (31, 32), and IgG and SIgA synergistically clear antigens and strengthen mucosal homeostasis (33, 34). The coordinated upregulation of these physical, chemical, and immune barrier components in DH suggests a sophisticated “defense network” that compensates for its relatively lower absorption efficiency, ensuring intestinal health and stress resistance under harsh breeding conditions.

A critical insight from this study is the functional trade-off and complementary adaptation between the two breeds: DHB prioritizes “nutrient absorption + oxidative stress resistance” to support rapid lean growth, while DH emphasizes “barrier integrity + immune competence” to enhance environmental adaptability and disease resistance. This functional differentiation is rooted in their genetic backgrounds and breeding objectives: DHB was selected for commercial traits (growth rate, meat yield), driving the optimization of metabolic and absorptive pathways; DH, as a traditional local breed, retains genetic loci associated with stress resistance and immune regulation, which are crucial for survival in extensive breeding systems (7, 12). Mechanistically, this trade-off may be mediated by the “intestinal-metabolic-immune axis”: in DHB, the activation of growth-related signaling pathways (e.g., PI3K-AKT) promotes intestinal epithelial proliferation (shallow crypts, high V/C ratio) and upregulates antioxidant enzymes to meet metabolic demands; in DH, the enhancement of TLR/MyD88 and NF-κB signaling pathways strengthens barrier function and immune responses, which may compete for metabolic resources with absorptive processes, leading to relatively lower V/C ratios (32, 35). This hypothesis is supported by previous studies showing that immune activation can redirect nutrient allocation from growth to defense, resulting in trade-offs between growth performance and disease resistance (4). Comparative analysis with other pig breeds further validates this functional differentiation pattern. For example, Tibetan pigs (a native breed) exhibit longer villi and stronger antioxidant capacity than DLY pigs (a commercial hybrid), but their immune barrier-related gene expression is also higher, reflecting adaptation to high-altitude stress (24); Meishan piglets (a local breed) have higher intestinal barrier function than crossbreds, consistent with DH's barrier advantage (22). However, DHB's unique combination of Duroc's growth potential and DH's basal intestinal function distinguishes it from other hybrids, as its antioxidant capacity is significantly higher than that of DH, highlighting the genetic contribution of Duroc to redox regulation (9, 21).

This study also has certain limitations. First, the mechanism underlying the functional trade-off between absorption/antioxidation and barrier/immunity remains to be elucidated at the molecular level—future studies should investigate key signaling pathways (e.g., PI3K-AKT, TLR/MyD88, NF-κB) and their crosstalk using multi-omics techniques (transcriptomics, proteomics, metabolomics). Second, the study only focused on the finishing stage; dynamic changes in intestinal function during critical periods (weaning) and their association with growth performance require further exploration. Third, the role of gut microbiota in mediating these functional differences was not addressed—short-chain fatty acids (SCFAs) produced by gut microbiota can regulate both intestinal barrier function and antioxidant capacity (36, 37), and future research should integrate microbiota analysis to clarify the host-microbe interactions underlying breed-specific intestinal traits. Despite these limitations, the core findings of this study still clearly reveal the divergent intestinal functional characteristics of DH and DHB shaped by genetic selection and environmental adaptation, and the identified breed-specific functional advantages provide a clear theoretical basis for the precision nutrition and healthy breeding of these two local pig breeds.

In conclusion, this study systematically reveals the breed-specific intestinal functional characteristics of DHB and DH: DHB's jejunum is specialized for efficient nutrient absorption and oxidative stress resistance, while DH's jejunum is adapted for robust barrier function and immune defense. These differences are the result of genetic selection and environmental adaptation, reflecting a functional trade-off in the intestinal-metabolic-immune axis. The findings not only deepen our understanding of the genetic and physiological mechanisms underlying intestinal function differentiation in local pig breeds but also provide targeted theoretical support for precision nutrition and breeding strategies: for DHB, dietary formulations should optimize nutrient density and balance to match its high absorptive capacity, while supplementing antioxidants (e.g., vitamin E, polyphenols) to enhance redox homeostasis; for DH, nutrition should focus on maintaining intestinal barrier integrity (e.g., supplementing glutamine, SCFAs precursors) to maximize its disease resistance potential. Additionally, the functional trade-off identified in this study offers a new perspective for hybrid breeding—integrating DHB's growth-related loci with DH's barrier/immune-related loci may yield new breeds with both high production performance and strong adaptability.

## 5 Conclusions

Analysis of intestinal structure shows that the jejunum of DHB have stronger nutrient absorption capacity; analysis of intestinal goblet cell number, intestinal barrier function gene expression level and immunoglobulin content shows that the jejunum of DH have stronger barrier function. The findings of this study fill the gap in research on the intestinal physiology and immune characteristics of local pig breeds in Yunnan. For the first time, it systematically reveals the divergent evolutionary features of jejunum function between DH and DHB, confirming breed-specific variations in intestinal morphology, antioxidant capacity, and barrier function. This provides new experimental data and research perspectives for elucidating the genetic regulatory mechanisms of pig intestinal function. Simultaneously, the study identifies the functional advantages of two local pig breeds, offering crucial theoretical foundations and practical support for precision nutritional regulation and healthy breeding of local pig breeds. In production practice, the high nutrient absorption capacity of DHB can be leveraged to optimize its dietary composition and digestibility, aligning with its rapid growth characteristics. Conversely, DH's superior intestinal barrier function and disease resistance can serve as high-quality parental resources for disease-resistant breeding. By combining DHB's growth and absorption advantages, hybrid selection can be conducted to develop new pig breeds with excellent growth performance and strong intestinal disease resistance. Additionally, the comprehensive evaluation method for local pig intestinal function established in this study provides a reference framework for the exploration of germplasm resources, functional characterization, and industrial utilization of other local livestock and poultry breeds.

## Data Availability

The original contributions presented in the study are included in the article/supplementary material, further inquiries can be directed to the corresponding authors.
